# Combination predicting model of traffic congestion index in weekdays based on LightGBM-GRU

**DOI:** 10.1038/s41598-022-06975-1

**Published:** 2022-02-21

**Authors:** Wei Cheng, Jiang-lin Li, Hai-Cheng Xiao, Li-na Ji

**Affiliations:** School of Traffic Engineering, Kunming University of Science and Technology, Kunming, 650000 China

**Keywords:** Civil engineering, Mathematics and computing, Scientific data

## Abstract

Tree-based and deep learning methods can automatically generate useful features. Not only can it enhance the original feature representation, but it can also learn to generate new features. This paper develops a strategy based on Light Gradient Boosting Machine (LightGBM or LGB) and Gated Recurrent Unit (GRU) to generate features to improve the expression ability of limited features. Moreover, a SARIMA-GRU prediction model considering the weekly periodicity is introduced. First, LightGBM is used to learn features and enhance the original features representation; secondly, GRU neural network is used to generate features; finally, the result ensemble is used as the input for prediction. Moreover, the SARIMA-GRU model is constructed for predicting. The GRU prediction consequences are revised by the SARIMA model that a better prediction can be obtained. The experiment was carried out with the data collected by Ride-hailing in Chengdu, and four predicted indicators and two performance indexes are utilized to evaluate the model. The results validate that the model proposed has significant improvements in the accuracy and performance of each component.

## Introduction

With the rapid economic development, the number of residents' vehicle ownership has increased year by year. Traveling demand has surged and traffic congestion is inevitable. Platforms such as Baidu and Gaud provide congestion index to guide travel. However, the existence of delay makes it unrealistic to avoid congestion in advance. Thus, it’s really important to predict the congestion index in real-time.

The limitation of collection technology makes the lack of traffic characteristics, which leads to the error of prediction. To solve the problem, it can be considered to enhance and generate features depend on original dataset. Yang Zhao et al.^1^ reported a hybrid LSTM-GCN model with three-way temporal features (SCLN-TTF), leveraging Long Short-Term Memory Network(LSTM) to extract temporal features, and graphing convolutional neural networks to extract spatial features; He et al.^2^ introduced a technique for prediction based on trees and deep learning (DL). The essay described a mean of feature generation, which is of great significance for feature extraction and generation. In a word, deep learning and tree based theory can infer new features with limited features in the dataset, and has the ability to create features. GRU is a typical representative in the field of deep learning, with the characteristics of simple structure and high efficiency.

With regard to the prediction technique, machine learning has the advantage of unnecessary assumptions or prior knowledge. It can automatically extract useful information from the dataset, which makes up for the shortcomings of traditional methods. Ensemble learning is an important branch of machine learning that has received widespread attention. It can combine multiple learners to reduce generalization errors, reduce the possibility of local optimization, and solve the problem of overfitting.

Fangjie Wang et al.^3^ developed multiple characteristics of bus line operation and the LightGBM algorithm were utilized to assign the weight of each factor to predict. Guoyan Xu et al.^4^ suggested an ensemble algorithm to construct a combined forecasting model of GRU and LightGBM to predict the water level, yet the model did not fully consider the characteristics. In^5^, the LSTM-SPRVM model and the fuzzy comprehensive evaluation-based method were leveraged to predict and rank the congestion, and a traffic congestion prediction and visualization framework based on machine learning and a fuzzy comprehensive evaluation-MF-TCPV were proposed. Note that the framework can visually observe the results of congestion prediction, and the accuracy is also preferable. Moreover, Ref ^6^ considered a prediction model based on LSTM-LGB, by weighting the consequences obtained by the LSTM and LGB models, the prediction outcomes manifested that the combined model can efficiently improve the accuracy.

By investigating the above-mentioned studies, there still exits few matters: (1) the limitation of single model; (2) timeliness is not considered when forecasting; and (3) only a single feature is considered.

This paper is motivated by the circumstance that previous methods have their own advantages and shortages, and a particular previous approach may not be able to accurately and timely predict traffic congestion with limited features in workday. For purpose of achieving more accurate and robust traffic congestion prediction, we elucidate a feature generation fusion prediction model (LightGBM-GRU) capable of predicting traffic congestion with a small number of features.

The original contributions of the proposal are as follows:

- The number of features is small, and the LightGBM is utilized to learn and enhance the expression of the original features.
- A deep learning algorithm (i.e., GRU) is used as feature generation to generate deeper feature interactions.
- GRU significantly reduces the computational complexity.
- Improvements in real-time and accuracy of prediction.

## Related works

### Feature generation

Feature generation is the process of generating new features based on existing features that enhance the representation of the original feature^7^. At present, the method of feature generation is mainly based on automatic learning. There are a host of paper illustrate the effect of generating features on prediction works. Steffen Rendle^8^ used the decomposition parameter to model all interactions between variables and utilized the decomposition parameters between feature variables to extract feature combinations. Hongtao Shi et al.^9^ discovered a new feature optimization approach based on deep learning and Feature Selection (FS) techniques. Jiang et al.^10^ first applied the GBDT strategy to automatically extract effective features and feature interactions. This essay presented a new approach for generating synthetic features to impose a prior knowledge about data representation. Moreover, there is a large amount of bibliographic^11–13^ that demonstrated the contribution of tree-based algorithm and deep learning to feature generation, which is enough to support the idea of this manuscript.

There are many different feature generation methods. FM is suitable for highly sparse data scenarios. In practical applications, it is limited by the computational complexity, and generally only second-order crossover features can be taken into account. The FS method only focuses on the improvement of classification performance and ignores the stability of selected feature subsets on traffic data changes. The method of deep learning has many parameters and complex structure. GBDT has few parameters and a fast training process, which can combat overfitting. But there is a dependency between weak learners, which makes it difficult to train data in parallel. Combining deep learning and tree-based approaches can combine the advantages of both approaches for feature generation.

### Prediction

As large dataset become more accessible, research on congestion prediction also tends to deep learning. Previous studies ^14–17^ proposed a combinatorial model for prediction, including GRU, LSTM, CNN, etc. The results manifest that the deep learning greatly improves the accuracy of traffic prediction. Compared to LSTM, GRU has a less complex structure and can be trained faster than LSTM^18^. Therefore, in order to reduce the complexity of the model, GRU is used instead of LSTM. Meanwhile, researches which combine tree-based and deep learning for prediction^19,20^ improve reference. Traffic congestion index has complex stochastic and nonlinear characteristics, and it reveals a similar seasonality and weekly trends. There are quite a few study^21–23^ consider cyclical factor, convert the volatility series into a stationary series to predict.

Predictive models can be summarized as statistical models and deep learning methods. Statistical models (ARIMA, Time series, etc.) are simple and effective in short-term prediction, but have higher complexity when there are more parameters to be estimated; deep learning methods (LSTM, CNN, etc.) have advantages in accuracy, and cannot completely solve the problems of gradient explosion and computational complexity. Combining the advantages of different algorithm models in prediction can make better predictions.

This study combines LightGBM and GRU to construct a feature generation model to predict congestion index. The tree-based theory and deep learning algorithm are used to enhance the expression of limited features. Furthermore, the seasonal GRU model aims to reduce complexity while ensuring accuracy when prediction.

## Methods

### Related definitions

The traffic congestion index is usually described by parameters such as flow and speed. Different areas and periods have different definitions. However, it is not necessarily a universal conception. Based on floating car data, the index of the traditional Traffic Performance Index (TPI) ranges from 0 to 10, and the degree of congestion is proportional to the value ^24^. In this paper, since the source of data is Didi, Travel Time Index (TTI) is leveraged. It’s commonly employed for navigation platform (i.e. Gaode, Didi and Baidu). Compared with TPI, TTI is more objective due to automatic evaluation. The definition of TTI is the ratio of free flow speed to actual speed within a certain period of time, and it can be obtained as:

1 $$TTI = \frac{{\mathop \sum \limits_{i = 1}^{N} \frac{{L_{i} }}{{V_{i} }} \cdot W_{i} }}{{\mathop \sum \limits_{i = 1}^{N} \frac{{L_{i} }}{{V_{{{\text{free\_i}}}} }} \cdot W_{i} }}$$

where $L_{i}$ is the length of the road section, $W_{i}$ is the road section weight, $V_{free\_i}$ is the free flow speed of the road section, and $V_{i}$ is the real-time speed of the road section. As shown in Eq. (1), time and speed are important character variable, having a correlation with congestion index. Among them, time has a greater impact on congestion, especially the huge difference between weekdays and holidays. For weekdays, residents travel time is concentrated and strongly regularity; for holidays, the residents flow is large, and the fluctuation of traffic flow is elusive. As the result, the object of this study is weekdays.

### Light gradient boosting machine

Boosting is one of the integrated algorithms. Depending on the loss function, there are different types of Boosting algorithms, as shown in Table 1.

**Table 1:** Development history of boosting algorithm.

Algorithm	Author & year
AdaBoosingt^25^	Freund & Schapire, 1996
Gradient boosting decision tree, GBDT^26^	Freidman, 2001
Gradient-boost AR ensemble learning algorithm, AREL^27^	YunLong Gao et al., 2011
eXtreme gradient boosting decision tree, XGBoost^28^	Tianqi Chen, 2016
Light gradient boosting machine, LightGBM^29^	Microsoft Corporation, 2017
Categorical boosting, CatBoost^30^	Liudmila Prokhorenkova et al., 2019
Deep decision tree transfer boosting, DTrBoost^31^	Shuhui Jiang et al., 2020

LightGBM is a lightweight algorithm based on the Gradient Boosting framework. It is currently a more advanced and mature methodology. Compared with XGBoost, it has the advantages of low memory, faster training efficiency, and higher accuracy.

**Figure 1:**
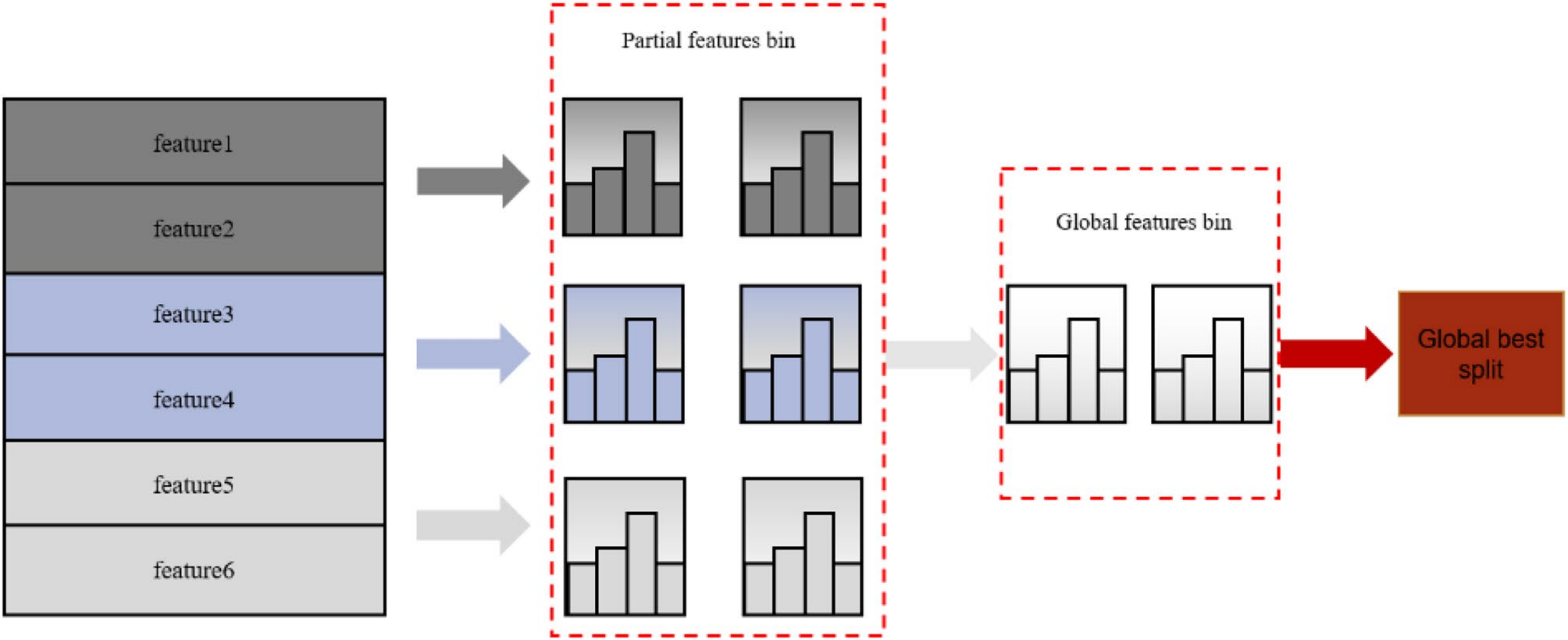
Light gradient hoist histogram algorithm principle.

These advantages are based on the histogram algorithm^32^, and the whole conversion process is shown in Fig. 1. The histogram algorithm converts each column of eigenvalues into a histogram, generates *k* bins according to the integer interval, and then places the eigenvalues in the bins of the corresponding interval, so that the bins <  < features, thereby reducing memory usage and calculation complexity.

Additionally, leaf-wise growth strategy with depth limitations can reduce the risk of overfitting while ensuring high efficiency. The process of constructing LGB is:

1. Initialize *n* decision trees and set the weight of the training set to $\frac{1}{n}$,
2. Training weak classifier,
3. Consider the weight of the weak classifier and update the weight,
4. Get the final classifier, see as:
  2 $$H_{T} (x) = \sum\limits_{t = 1}^{T} {h(x,\theta_{t} )}$$
  where $h(x,\theta_{t} )$ is the prediction result after training a regression tree,$\theta_{t}$ is the output of the decision tree model of input *x*, which is the parameter of decision tree *t*.

### Gated recurrent unit

**Figure 2:**
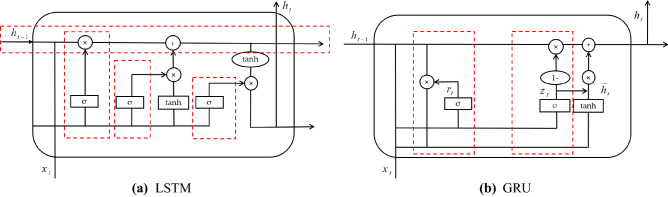
The structure of LSTM and GRU.

GRU^33^ is a variant of LSTM, similar to the goals achieved by LSTM, but its complexity is lower than that of LSTM. The structure of the two algorithms are compared in Fig. 2.

LSTM maintains 4 sets of parameters, corresponding to input gates, output gates, forget gates and cell states. The total number of parameters is shown as:

3 $$Total\_Number = 4*Hidden\_size*\left( {Input\_size + Bias + Output\_size} \right)$$

When it is not a project, $Output\_size$ and $Hidden\_size$ are the same. Thus $Output\_size + Hidden\_size$ is $concat[h_{t - 1} ,x]$. Suppose there is only one hidden unit, the total number become $4\left( {nm + n^{2} + n} \right)$. The complexity of LSTM is $O\left( {4\left( {nm + n^{2} + n} \right)} \right)$.

As Fig. 2 (b), The operating process of GRU can be calculated, as:

4 $$r_{t} = \sigma \left( {W_{r} * \left[ {h_{t - 1} ,x_{t} } \right] + b_{r} } \right)$$

5 $$z_{t} = \sigma \left( {W_{t} * \left[ {h_{t - 1} ,x_{t} } \right] + b_{z} } \right)$$

6 $$\tilde{h}_{t} = \tanh \left( {W_{{\tilde{h}}} * \left[ {r_{t} \cdot h_{t - 1} ,x_{t} } \right] + b_{{\tilde{h}}} } \right)$$

7 $$h_{t} = \left( {1 - z_{t} } \right) \cdot h_{t - 1} + z_{t} \cdot \tilde{h}$$

where $r_{t}$ represents the reset gate, $z_{t}$ represents the update gate, $h_{t}$ represents the final output, $x_{t}$ is the current input, and obtains the data after the control signal, i.e., $\mathop h\limits^{\sim }_{t}$, and both $\tanh ( \cdots )$、$\sigma ( \cdots )$ are activation functions.

GRU contains 2 sets of parameters, corresponding to reset gates and update gate. Similarly, The complexity of GRU is $O\left( {2\left( {nm + n^{2} + n} \right)} \right)$.

Compared with LSTM, the advantage of GRU lies in the simple structure and easy to train. Besides, the gated structure can well solve the problems of gradient disappearance and gradient explosion. Considering the real-time problem of prediction, a simpler gated recurrent unit structure is adopted.

### SARIMA

When constructing a forecasting model, consider the periodicity of time series data is necessary. The Seasonal Autoregressive Integrated Moving Average (SARIMA) model can consider the influence of seasons, monthly cycles, weekly cycles, etc. on the data. When predicting the congestion, more accurate results can be obtained with the SARIMA. According to the prediction consequences in the SARIMA model, the unit node ratio sequence of the period and trend information is extracted, and the results are revised^34^. The process can be expressed as:

8 $$TTI_{p} = X_{total} \cdot \theta_{i}$$

9 $$X_{total} = \sum X$$

10 $$\theta_{i} = \frac{{S_{i} }}{{\sum {S_{i} } }}$$

where $X$ represents the value of the preliminary prediction, $TTI_{p}$ refers to the final predicted value of the congestion index, and $\theta_{i}$ refers to the proportional sequence of the node $S_{i}$ per unit time extracted from the outcome of the SARIMA model.

### Combination model

#### Feature generation model based on LightGBM-GRU

Traditional decision tree algorithms cannot learn feature combinations that rarely appear in the training data. Although deep learning means can extract feature combinations through the inner product of hidden vectors, they have limitations in extracting low-level feature combinations^35^. Therefore, this research combines tree-based and deep learning methods. Not only can the expression ability of limited features be improved, but also features can be generated to achieve superior accuracy. The steps of the prediction model generated based on LightGBM-GRU features can be summarized, as:

1. Data preprocessing. Cleaning and filtering the original data.
2. Processing time characteristics, converting data in date format into timestamps to facilitate subsequent preparations for time grouping and difference calculations.
3. Process digital features, standardize and normalize digital features such as speed and congestion index, and unify the scales of various features to make them comparable.
4. Initially set the learning rate, number of estimators, tree depth and other parameters of LightGBM to train the model.
5. Enter the LightGBM processed features into the GRU. Set the parameters of the GRU, such as the number of neurons, time step, activation function, etc., and generate features.
6. Finally, the resulting feature matrix is fused.

### Combination forecasting model based on SARIMA-GRU

First, SARIMA model is constructed to predict the previously generated features; second, the unit node ratio sequence of period and trend information based on the output of the SARIMA model is extracted; third, GRU is used to make preliminary predictions on the congestion index; fourth, the period information extracted by SARIMA is applied to revise the preliminary results that obtained by GRU.

**Figure 3:**
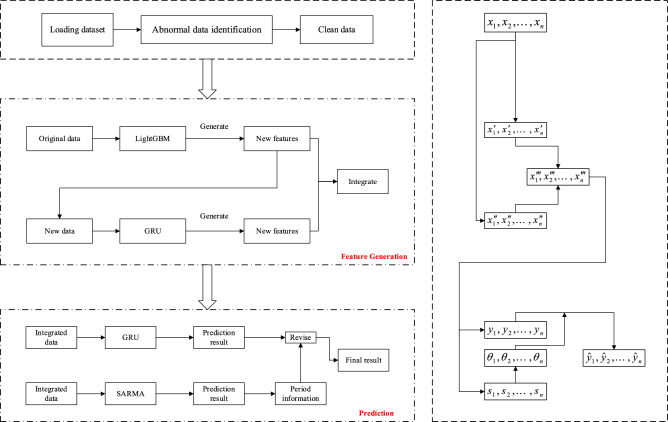
Frame diagram of the model.

The whole process framework is shown in Fig. 3.

## Results and analysis

### Experimental data

The data in this article comes from the “Gaia Open Dataset” of Didi. The dataset is quoted to contain the trajectory data of the Didi express car platform in Chengdu and Xi'an in 2018. The traffic congestion index and average driving speed in the area is also contained (Data Sources: https://outreach.didichuxing.com/research/opendata/). This dataset divides the traffic index into 4 levels, which can be utilized as the basis for classification. Table 2 list the specific standards.

**Table 2:**
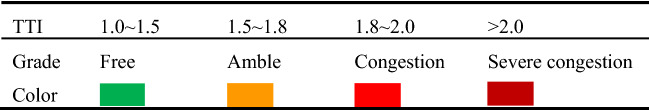
The classification of traffic congestion index.

Traffic congestion is highly correlated with time, and the congestion index calculated by speed can describe the traffic state. According to^36^, traffic volume has timely distribution characteristics, which are closely related to hour, day, and month. This study leverage data from weekdays in Chengdu, and the platform to implement the model is Python 3.7.0.

Preprocessing of data is a critical step before building and training models. First, identify missing and exception value and replace them with averages. Then, normalization makes data comparable. Ren et al.^37^ utilized the Eq. (11) to normalize.

11 $$x^{ * } = \frac{{x - x_{\min } }}{{x_{\max } - x_{\min } }}$$

where $x_{\min }$, $x_{\max }$ are the minimum and maximum values of the current eigenvalues respectively, $x^{*}$ representing the standardized eigenvalues, and $x$ is the original eigenvalues.

### Model evaluation indicators

To evaluate the effect of the model, multiple evaluation indicators are employed to analyze the results of the experiment, which are the RMSE, the MSE, the MAE, and the MAPE^38^. It can be expressed as:

12 $$MSE(y,\hat{y}) = \frac{1}{n}\mathop \sum \limits_{i = 0}^{n - 1} \left( {y_{i} - \hat{y}_{i} } \right)^{2}$$

13 $$RMSE(y,\hat{y}) = \sqrt {\frac{1}{n}\sum\limits_{i = 0}^{n - 1} {\left( {y_{i} - \hat{y}_{i} } \right)}^{2} }$$

14 $$MAE(y,\hat{y}) = \frac{1}{n}\mathop \sum \limits_{i = 0}^{n - 1} \left| {y_{i} - \hat{y}_{i} } \right|$$

15 $$MAPE(y,\hat{y}) = \frac{1}{n}\mathop \sum \limits_{i = 0}^{n - 1} \left| {\frac{{y_{i} - \hat{y}_{i} }}{{y_{i} }}} \right|$$

16 $$Accuracy\left( \% \right) = \left( {1 - MAPE} \right)*100$$

## Results and analysis

Considering seasonal factors and weekly periodicity, the first week of the four quarters is selected from the original data as the research object, namely 2018.3.5 ~ 3.9, 2018.6.4 ~ 6.8, 2018.9.3 ~ 9.7, 2018.12.3 ~ 12.7.

**Figure 4:**
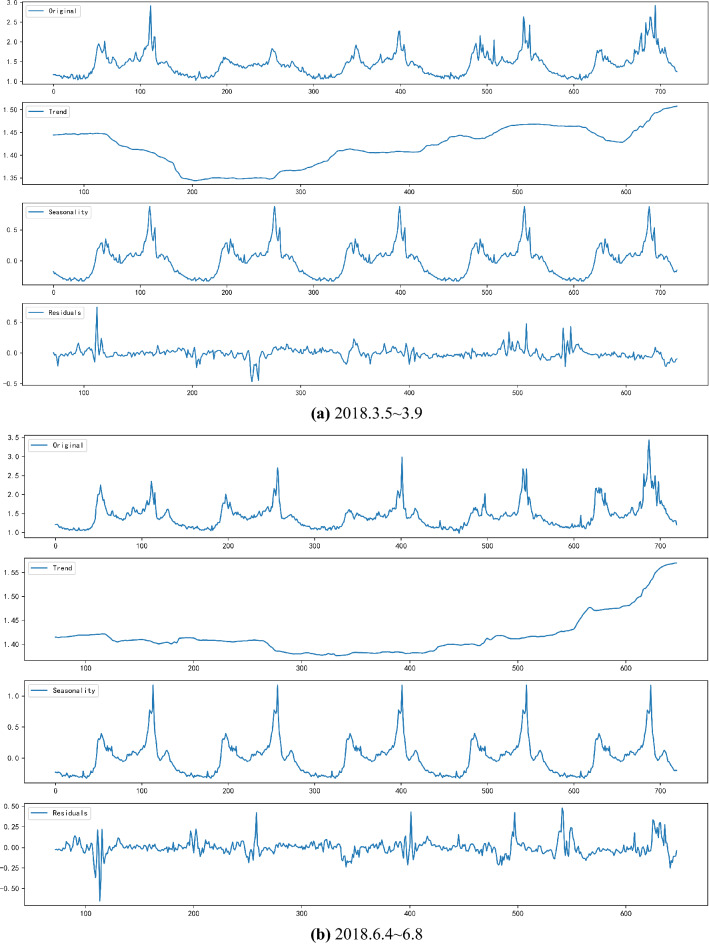

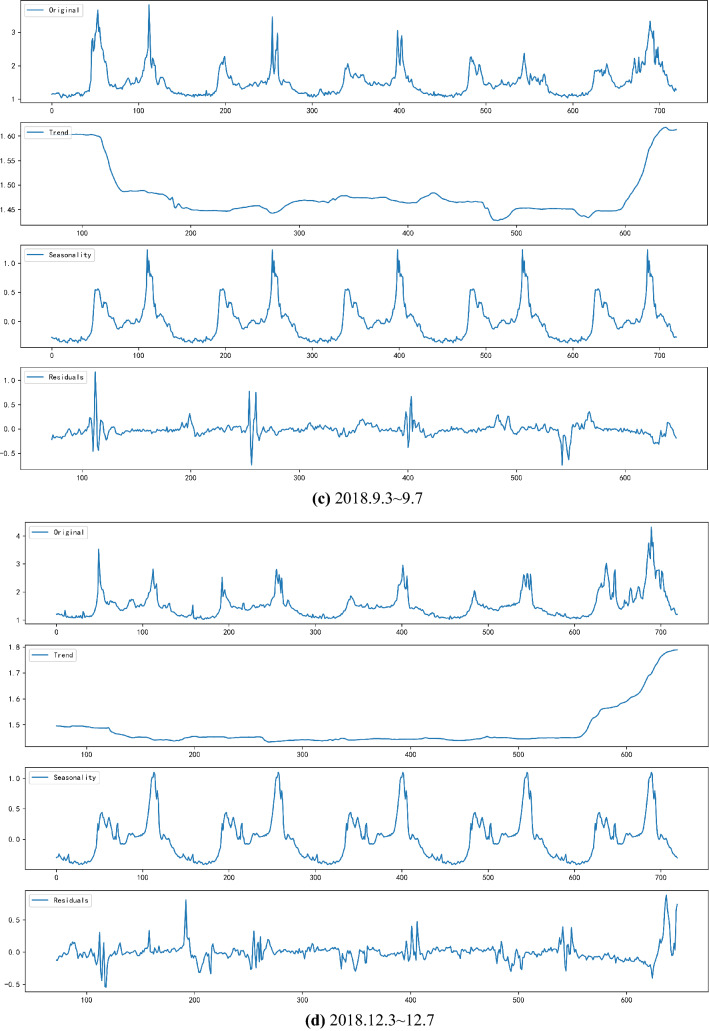
The decomposition result.

First, SARIMA is used to decompose the original data, and the time series data is separated into different components: Trend, Seasonality and Random Residuals. The decomposition output is shown in Fig. 4.

From the Fig. 4, traffic congestion changes periodically over the course of a day. On weekdays of the week, the change varies slightly from Monday to Friday. In the Trend chart, it can be observed that the congestion trend from Tuesday to Thursday during the week is relatively flat, and the congestion index on Monday and Friday is relatively large. Shown in the seasonality graph is a stationary sequence that breaks down seasonal factors. The last subgraph is the seasonal factor of the division release and is a fluctuating value. To draw a conclusion, changes of the traffic congestion index are cyclical, and SARIMA is utilized to decompose seasonal characteristics. It is a crucial step before a formal prediction is made.

**Figure 5:**
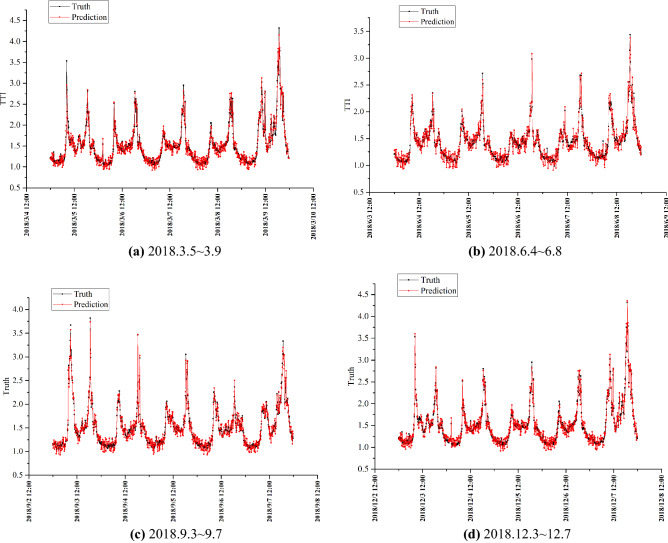
The model predicts results over four working days.

**Figure 6:**
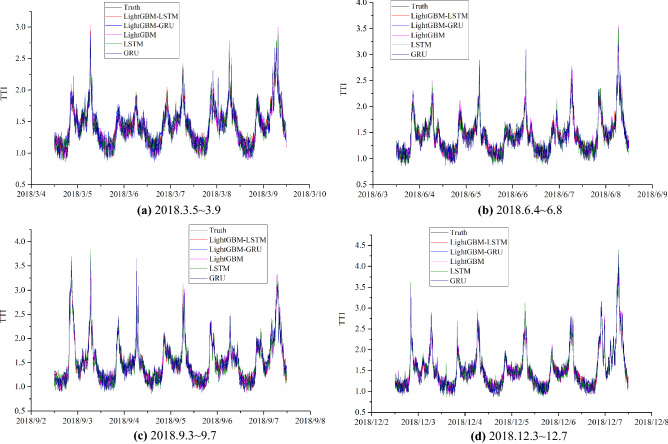
Comparison of model results.

Next, using 10 min as the time granularity, construct a seasonal ARIMA model to process the data, and then revise the GRU results to get the final value which shown in Fig. 5. The comparison with the prediction results of other advanced technologies is shown in Fig. 6.

**Figure 7:**
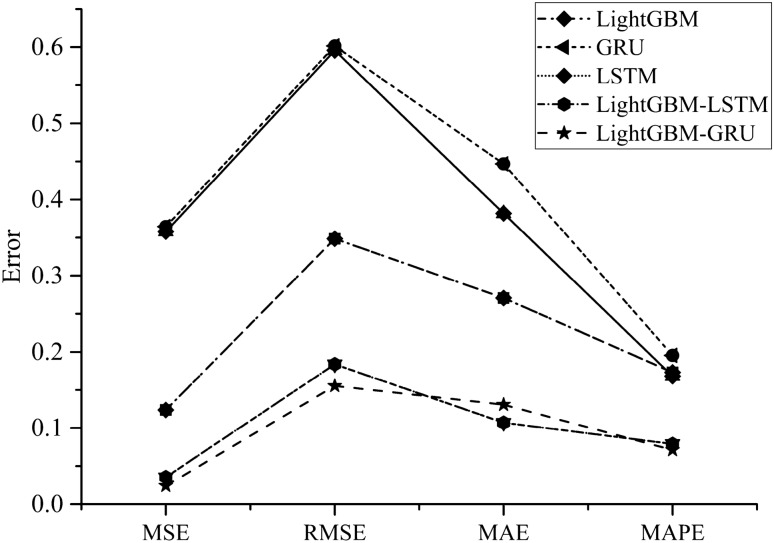
Error comparison.

In Fig. 5, the predicted value is similar to the curve of the actual value, and the predicted result is accurate. The results indicate that the congestion index changes regularly: The peaks are obvious in the morning and evening during the week, and the peaks are higher on Monday and Friday. Usually, the morning on Monday is more congested, and the evening on Friday is more congested and lasts longer. In Fig. 6, several methods are consistent in the prediction of the overall trend, but there are significant fluctuations in the shorter period of time. In order to quantitatively compare the results of LightGBM, GRU, LSTM and LightGBM-LSTM, error calculation is indispensable. The values of errors are shown in Table 3 and Fig. 7.

**Table 3:** Prediction error.

	MSE	RMSE	MAE	MAPE	Accuracy1 (%)	Accuracy2 (%)
LightGBM	0.124	0.349	0.271	0.173	82.7	80.6
GRU	0.365	0.601	0.447	0.196	80.5	77.6
LSTM	0.358	0.596	0.382	0.168	83.2	82.1
LightGBM-LSTM	0.036	0.184	0.107	0.079	92.1	90.1
LightGBM-GRU	0.024	0.155	0.128	0.054	92.9	90.1

Mean Absolute Percent Error (MAPE) is used to calculate the accuracy of model comparison and evaluation in different time ranges^39^. In the table above, Accuracy1 represents the accuracy of the prediction results after feature generation, and Accuracy2 represents the accuracy of the direct prediction without feature generation. It is obvious that the accuracy of the predicted values processed by feature generation processing is 1% to 2% higher. This proves the effectiveness of the feature generation method. Meanwhile, compared with other models, the model in this paper has the best accuracy and is up to 10% higher in accuracy than other models. The accuracy of LightGBM-LSTM is similar to that of the LightGBM-GRU, only 0.8% lower, and there is not much difference.

**Figure 8:**
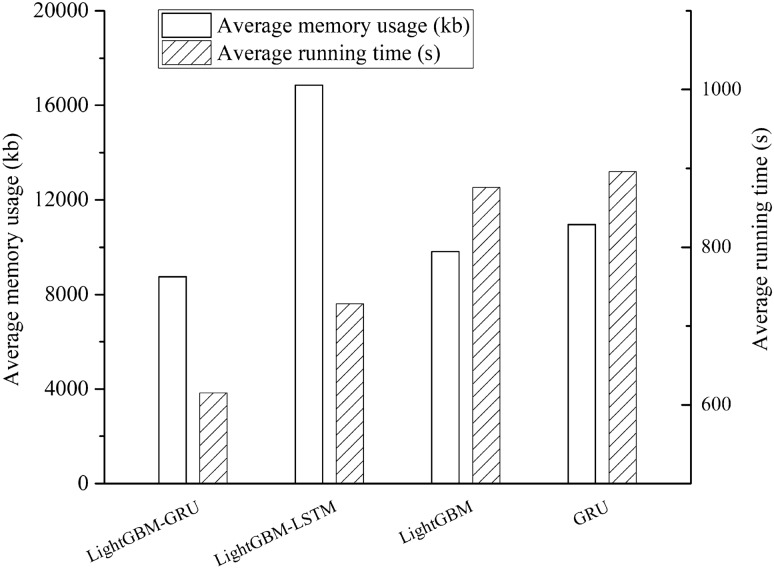
Performance comparison.

In addition, the performance of the mode is also an excellent part of the evaluation model. The complexity of the model is measured by the average running time and the average occupied memory, so that the performance characteristics of the model can be analyzed intuitively. The results are shown in Fig. 8 and Table 4.

**Table 4:** Model performance value.

	LightGBM-GRU	LightGBM-LSTM	LightGBM	GRU	LSTM
Average running time(s)	615	728	876	896	1054
Average memory usage(kb)	8754	16,850	9812	10,961	18,325

From the above chart, we can see the model has advantages in terms of running time and memory consumption. Compared with LightGBM-LSTM with similar accuracy, the running time of this model is reduced by 15.52% and memory consumption by 48.05%. For the prediction of the congestion index, real-time performance is extremely important. A faster calculation speed can reduce the delay of the prediction, and a smaller memory footprint is of great significance for optimizing the algorithm. For LSTM models similar to GRU, both have relatively small prediction errors, but the GRU model is excellent in terms of complexity and memory consumption. On the whole, the model has a preferable performance.

## Discussion

This study uses the weekday data of Didi in Chengdu to construct and successfully test the traffic congestion index prediction model. The consequences manifest that based on the prediction accuracy and algorithm performance, the model can predict congestion index more accurately.

The results demonstrate that the congestion index changes cyclically. Within one day, the congestion shows a double-peak change. Within one weekday, the peak of congestion from Tuesday to Thursday is lower than that on Monday and Friday. Taking this regular change factor into consideration, the SARIMA model is used to revise the outcome. The final results imply that the accuracy of the GRU and LSTM methods is similar in prediction, and both can reach an accuracy of more than 90%; in terms of performance, the memory occupied by LSTM is 1.92 times that of GRU, and the calculation speed is 1.18 times that of GRU. Therefore, it helps the congestion prediction model to obtain a better accuracy and the experiments have proved its robustness and efficiency in weekday.

### Conclusions and future research directions

In this paper, LightGBM-GRU is used to generate features, and SARIMA-GRU is used to predict congestion index. Under the framework of the construction, the following two aspects can be solved: One is that it provides a strategy for dealing with fewer features, solving the problem of single features; and the other is that it fully considers the impact of data periodicity and solves the problem of predicting time-period factors interfering with prediction.

At the same time, while improving the prediction accuracy, it also reduces the complexity and memory usage of the model. The main conclusions are as follows:

• The LightGBM-GRU solved the lake of features in the original data and built a model for feature generation. LightGBM and GRU are utilized to analyze and train the original features of the dataset to generate feature matrix.

• The combined model took the seasonality of weekdays into consider. As long as improved accuracy and computing speed. By comparing with other models, it can effectively modify the prediction results, so that the accuracy, stability and running speed are improved.

The results confirmed that the feature generation method can effectively predict when the feature is limited. However, there is also few shortcomings. In future research, the factors that affect the congestion index, such as weather, environment, and driver's psychological characteristics, should be considered more comprehensively. When data acquisition allows, try to expand the scope of research to study more hidden information. Furthermore, while adding features, the accuracy and complexity of the algorithm should also be considered.
